# Management and outcome trends in type 2 myocardial infarction: an investigation from the SWEDEHEART registry

**DOI:** 10.1038/s41598-023-34312-7

**Published:** 2023-05-03

**Authors:** K. M. Eggers, T. Baron, A. R. Chapman, A. Gard, B. Lindahl

**Affiliations:** 1 Department of Medical Sciences, CardiologyUppsala University, 751 85 Uppsala, Sweden; 2 Uppsala Clinical Research Center, Uppsala University, Uppsala, Sweden; 3 BHF Centre for Cardiovascular Science, University of Edinburgh, Edinburgh, UK

**Keywords:** Cardiology, Cardiovascular diseases

## Abstract

Despite poor prognosis, patients with type 2 myocardial infarction (MI) tend to be underdiagnosed and undertreated compared to those with type 1 MI. Whether this discrepancy has improved over time is uncertain. We conducted a registry-based cohort study investigating type 2 MI patients managed at Swedish coronary care units (n = 14,833) during 2010–2022. Multivariable-adjusted changes (first three vs last three calendar years of the observation period) were assessed regarding diagnostic examinations (echocardiography, coronary assessment), provision of cardioprotective medications (betablockers, renin–angiotensin–aldosterone-system inhibitors, statins) and 1-year all-cause mortality. Compared to type 1 MI patients (n = 184,329), those with type 2 MI less often had diagnostic examinations and cardioprotective medications. Increases in the use of echocardiography (OR 1.08 [95% confidence interval 1.06–1.09]) and coronary assessment (OR 1.06 [95% confidence interval 1.04–1.08]) were smaller compared to type 1 MI (p_interaction_ < 0.001). The provision of medications did not increase in type 2 MI. All-cause mortality rate in type 2 MI was 25.4% without temporal change (OR 1.03 [95% confidence interval 0.98–1.07]). Taken together, the provision of medications and all-cause mortality did ot improve in type 2 MI despite modest increases in diagnostic procedures. This emphasizes the need of defining optimal care pathways in these patients.

## Introduction

Type 2 myocardial infarction (MI) is defined as acute ischemic myocardial injury due to oxygen supply/demand mismatch caused by a condition other than coronary plaque disruption or coronary intervention^1^. Compared to patients with type 1 MI, those with type 2 MI tend to be older and more frequently suffer from comorbidities, both cardiovascular and non-cardiovascular^2–7^. Outcomes after type 2 MI are poor; up to one third of the population will die within one year from the event^5,6^. Paradoxically however, type 2 MI patients tend to be underdiagnosed and undertreated^2–7^. This likely reflects clinical challenges related to the heterogeneity of underlying etiologies, the distinction of non-ischemic myocardial injury in patients with complex conditions, the presence of increasing age and co-morbidites, and the lack of evidence from prospective randomised controlled trials to guide treatment.

The concept of type 2 MI was introduced in 2007 together with the publication of the Universal Definition of MI^1^. It is conceivable that an increased awareness of type 2 MI as a high-risk condition may have influenced patient management through increases in the use of diagnostic examinations or of cardioprotective medications. This could plausibly have led to changes in outcomes for patients with type 2 MI. To investigate this issue, longitudinal information obtained over a sufficiently long period is needed. We here present registry-based data exploring changes in management patterns and all-cause mortality for a large cohort of type 2 MI patients who had been admitted during a 11-year period to Swedish coronary care units (CCU).

## Material and methods

### Study population

This study is part of the TOTAL-AMI (Tailoring Of Treatment in All comers with Acute Myocardial Infarction) project^8^. The primary aim of TOTAL-AMI is to study the mechanisms and implications of different MI subtypes^1^ and comorbidities (e.g. chronic obstructive pulmonary disease, atrial fibrillation, renal dysfunction) in MI. TOTAL-AMI uses data from SWEDEHEART (Swedish Web-system for Enhancement and Development of Evidence-based care in Heart disease Evaluated According to Recommended Therapies) which is a Swedish nationwide registry prospectively collecting data from patients admitted to CCUs or other specialized facilities because of suspected acute coronary syndrome. SWEDEHEART provides information on patient demographics, medical history, symptoms, physical and ECG findings upon admission, blood test results, in-hospital management, and discharge medications. Upon hospital admission, patients receive information about the registry, have the right to deny participation and get their data erased upon request. Written informed consent is not required according to Swedish law.

The cohort of interest for the present study included all type 2 MI patients admitted between January 1, 2010 and February 13, 2022. For comparative purposes, we also considered type 1 MI patients who had been hospitalized during the same period. All patients had been discharged with I21 or I22 (International Classification of Diseases, 10th revision, Clinical Modification) as primary diagnostic code.

All data had been made pseudonymized before the statistical analyses. The study was conducted according to the principles of the 1975 Declaration of Helsinki and had been approved by the Regional Ethical Review Board in Stockholm (2012/60–31/2).

### Diagnostic classification

The diagnoses recorded in SWEDEHEART are set by the treating physicians at each respective hospital. Since 2010, the use of the Universal Definition including its subclassifications^1^ is recommended within the SWEDEHEART framework^9^. Completeness of this information has gradually increased in the registry from 33.4% in 2010 to almost 100% from 2012 onwards (Supplemental Fig. 1).

### Investigated medical interventions

We investigated the annual rates of various medical interventions considered as quality measures for the management of type 2 MI: in-hospital echocardiography, in-hospital coronary assessment (invasive coronary angiography, treadmill test, myocardial perfusion scintigraphy, stress echocardiography), and provision of cardioprotective medications upon discharge: betablockers, renin–angiotensin–aldosterone-system (RAAS)-inhibitors or statins. For discharge medications, we focused on patients with coronary artery disease (CAD) since such treatments are strongly advised in these patients^10–12^. CAD was defined as previous MI, previous coronary revascularization, and coronary stenosis ≥ 50% upon invasive angiography or a pathologic non-invasive test performed during the hospitalization. We did not consider antiplatelet or anticoagulatory medications since decisions on treatment and treatment duration tend to be highly individualized in type 2 MI, depending on preexisting comorbidities, bleeding risk and the presence or absence of specific triggering conditions.

Since management decisions in type 2 MI might have been affected by specific comorbidities or contraindications, the following intervention-specific exclusion criteria were applied:

- Total cohort: dementia;
- Coronary assessment: ST-elevation upon admission, hemoglobin < 80 g/L;
- Discharge medication with betablockers: heart rate < 50/min upon admission;
- Discharge medication with RAAS-inhibitors: estimated glomerular filtration rate (eGFR; CKD-EPI equation) < 20 mL/min/1.73m^2^, left-ventricular ejection fraction > 0.50 in patients without concomitant diabetes, hypertension or known heart failure.

### Prognostic evaluation

The prognostic outcome was 1-year all-cause mortality. Information on mortality until end of May 5, 2022 was obtained from the Swedish Population Registry.

### Statistical analysis

All continuous variables were skewed and are reported as medians with 25th and 75th percentiles. Categorical variables are expressed as frequencies and percentages.

Changes in management were assessed using multivariable logistic regressions using the first three vs last three calendar years of the observation period (i.e. 2010–2012 vs. 2020–2022) as explanatory covariates. Adjustment was made for hospital, sex, age, current smoking, diabetes, previous MI, previous coronary revascularization, previous heart failure, previous stroke, atrial fibrillation upon admission, chronic obstructive pulmonary disease, previous or present cancer and peripheral artery disease. Subanalyses were conducted in men and women, respectively. To ensure the validity of our results, two sensitivity analyses were applied:

- Restricting the analyses to patients aged < 80 years since management decisions tend to be highly individualized in patients at higher ages;
- Restricting the analyses to patients with a first-time admission since specific management decisions could have been based on information obtained during a previous hospitalization.

Interaction terms were added to the fully adjusted models for comparisons of management patterns in type 2 MI patients with and without CAD, female and male type 2 MI patients and between patients with type 2 MI and type 1 MI.

Changes in all-cause mortality were investigated using the same approach with additional adjustment for in-hospital coronary revascularization. Kaplan–Meier curves were plotted to illustrate the cumulative incidence of all-cause mortality per admission year with differences calculated using the Log-rank test.

No imputation was performed in case of missing data. In all tests, a two-sided *p*-value < 0.05 was considered significant, apart from the interaction analyses where significance was reached at a *p* value < 0.10. The software package SPSS 27.0 (SPSS Inc., Chicago, IL) was used for the analyses.

## Results

**Figure 1:**
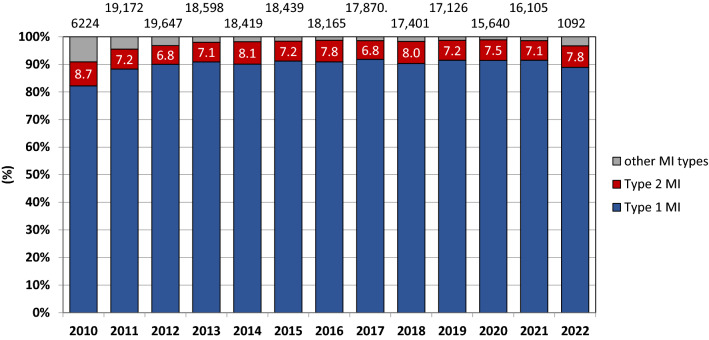
Annual prevalences of different MI types. The annual prevalences of type 2 MI are presented within the respective bars. The total numbers of patients with available information on MI type are presented on top of each bar. *MI* myocardial infarction.

A total of 203,891 patients had been admitted for MI during the observation period. Of these, type 1 MI accounted for 184,329 (90.4%) admissions, type 2 MI for 15,071 (7.4%) admissions and type 3–5 MI for 4498 (2.2%) admissions (Fig. 1). Following exclusion of 1687 admissions of patients with dementia, a total of 14,883 admissions for type 2 MI remained in the dataset. Of these, 7751 (52.1%) admissions occurred for patients with CAD. Following intervention-specific exclusions, trends in coronary assessment were evaluable in 13,383 type 2 MI admissions, and trends in the provision of betablockers and RAAS-inhibitors in 13,819 and 7919 type 2 MI admissions, respectively. Information on clinical characteristics and medical interventions is presented in Table 1.

**Table 1:** Clinical characteristics in patients with type 2 MI.

Demographics	Type 2 MI (n = 14,883)		Type 1 MI (n = 182,879)	
		Missing data		Missing data
Age (years)	78 (69–85)	–	72 (63–80)	–
Men	7494 (50.4%)	–	122,773 (67.1%)	–
Risk factors				
Current smoking	1859 (12.5%)	10	35,191 (19.2%)	53
Hypertension	9523 (64.0%)	8	104,346 (57.1%)	90
Diabetes	4080 (27.4%)	11	40,282 (22.0%)	131
Hyperlipidemia	6631 (44.6%)	18	62,622 (34.3%)	257
Body mass index (kg/m^2^)	25.8 (23.1–29.2)	1393	26.6 (24.1–29.7)	10,191
eGFR (mL/min/1.73m^2^)	61.5 (41.2–82.0)	630	76.3 (56.8–90.1)	5489
Comorbidities				
Previous MI	5563 (37.4%)	13	51,391 (28.1%)	118
Previous PCI/CABG	4082 (27.5%)	13	44,286 (24.2%)	121
Heart failure	2754 (18.5%)	13	18,105 (9.9%)	131
Atrial fibrillation (adm.)	3777 (25.4%)	40	16,626 (9.1%)	538
Previous stroke	1904 (12.8%)	14	14,736 (8.1%)	144
Peripheral artery disease	1752 (11.8%)	–	11,654 (6.4%)	–
COPD	2538 (17.1%)	–	13,942 (7.6%)	–
Previous/present cancer	1247 (8.4%)	–	8229 (4.5%)	–
Diagnostic procedures				
Echocardiography	9735 (65.4%)	–	149,386 (81.9%)	–
Coronary assessment	5301 (39.6%)	–	89,681 (79.5%)	–
Discharge medications*				
Betablockers	10,814 (78.3%)	–	144,893 (85.9%)	–
RAAS–inhibitors	5990 (75.6%)	–	95,643 (87.3%)	–
Statins	9296 (66.1%)	–	158,811 (91.1%)	–
Discharge medications in patients with CAD*				
Betablockers	6183 (85.5%)	–	not assessed	–
RAAS–inhibitors	3539 (78.0%)	–	not assessed	–
Statins	5632 (76.5%)	–	not assessed	–

*Data from hospital survivors (all type 2 MI: n = 14,116; type 2 MI with CAD: n = 7391; type 1 MI: n = 174,866).

Data are given as numbers with percentages, and as medians with 25th and 75th percentiles.

*MI* Myocardial infarction; *eGFR* Estimated glomerular filtration rate; *PCI* Percutaneous coronary intervention; *CABG* Coronary artery bypass grafting; adm: admission; *COPD* Chronic obstructive pulmonary disease; *RAAS* Renin–angiotensin–aldosterone-system; *CAD* coronary artery disease.

In patients with type 2 MI, significant temporal changes in the rates of echocardiography and coronary assessment by + 8% and + 6%, respectively were noted following multivariable adjustment (Table 2; Supplementary Fig. S2). These increases were similar in men and women, as indicated by non-significant interaction terms (Supplementary Table S1). The sensitivity analyses did not provide different results (Supplementary Table S2). However, the temporal increases in examination rates were smaller as noted for type 1 MI (echocardiography: odds ratio 1.13 [95% confidence interval 1.13–1.14]; p _interaction_ < 0.001; coronary assessment: odds ratio 1.12 [95% confidence interval 1.11–1.13]; p _interaction_ < 0.001).

**Table 2:** Temporal management changes in patients with type 2 MI.

	Crude data			Multivariable logistic regression results		
	2010–2012	2020–2022	Δ	n	OR (95% CI)	*p* value
Examinations						
Echocardiography	1908 (59.1%)	1737 (73.1%)	+ 14.0%	5242	1.08 (1.06–1.09)	< 0.001
Coronary assessment	979 (34.2%)	1058 (49.6%)	+ 15.4%	4678	1.06 (1.04–1.08)	< 0.001
Discharge medications						
Betablockers	2443 (82.2%)	1622 (73.4%)	− 8.8%	4866	0.94 (0.92–0.95)	< 0.001
RAAS-inhibitors	1176 (77.7%)	1043 (75.6%)	− 2.1%	2887	0.97 (0.95–1.00)	0.021
Statins	2018 (66.3%)	1560 (69.2%)	+ 2.9%	4958	0.99 (0.97–1.01)	0.992
Discharge medications in type 2 MI with CAD						
Betablockers	1433 (86.8%)	891 (82.5%)	− 4.3%	2563	0.96 (0.93–0.99)	0.004
RAAS-inhibitors	743 (79.7%)	564 (77.4%)	− 2.3%	1656	0.97 (0.94–1.00)	0.060
Statins	1279 (75.7%)	888 (80.5%)	+ 4.8%	2614	1.00 (0.97–1.03)	0.986

Multivariable logistic regressions were adjusted for hospital, sex, age, current smoking, diabetes, hypertension, hyperlipidemia, congestive heart failure, previous myocardial infarction, previous percutaneous coronary intervention or coronary artery bypass grafting; previous stroke, atrial fibrillation upon admission, chronic obstructive pulmonary disease, previous or present cancer, peripheral vascular disease, estimated glomerular filtration rate and admission years (2010–2012 vs. 2020–2022). *OR* odds ratio; *CI* confidence interval; *RAAS* renin–angiotensin–aldosterone-system; *CAD* coronary artery disease.

The provision of cardioprotective medications decreased in type 2 MI by 1–6% in adjusted models (Table 2, Supplementary Fig. S3). The sensitivity analyses yielded similar results (Supplementary Table S2) apart for patients with a first-time admission in whom neutral temporal trends were seen. Prescription rates were generally higher in type 2 MI patients with CAD compared to those without. However, the temporal trends were similar in both cohorts (Supplementary Table S3) as also noted when men and women were assessed separately (Supplementary Table S1). Treatment patterns differed to type 1 MI where an increase in statin prescription was noted (odds ratio 1.05 [95% confidence interval 1.05–1.06; p _interaction_ < 0.001]), a stronger decrease in betablocker prescription (odds ratio 0.88 [95% confidence interval 0.88–0.89; p _interaction_ < 0.001]) and no change in the prescription of RAAS-inhibitors (odds ratio 1.00 [95% confidence interval 0.99–1.00; p_interaction_ = 0.012]).

**Figure 2:**
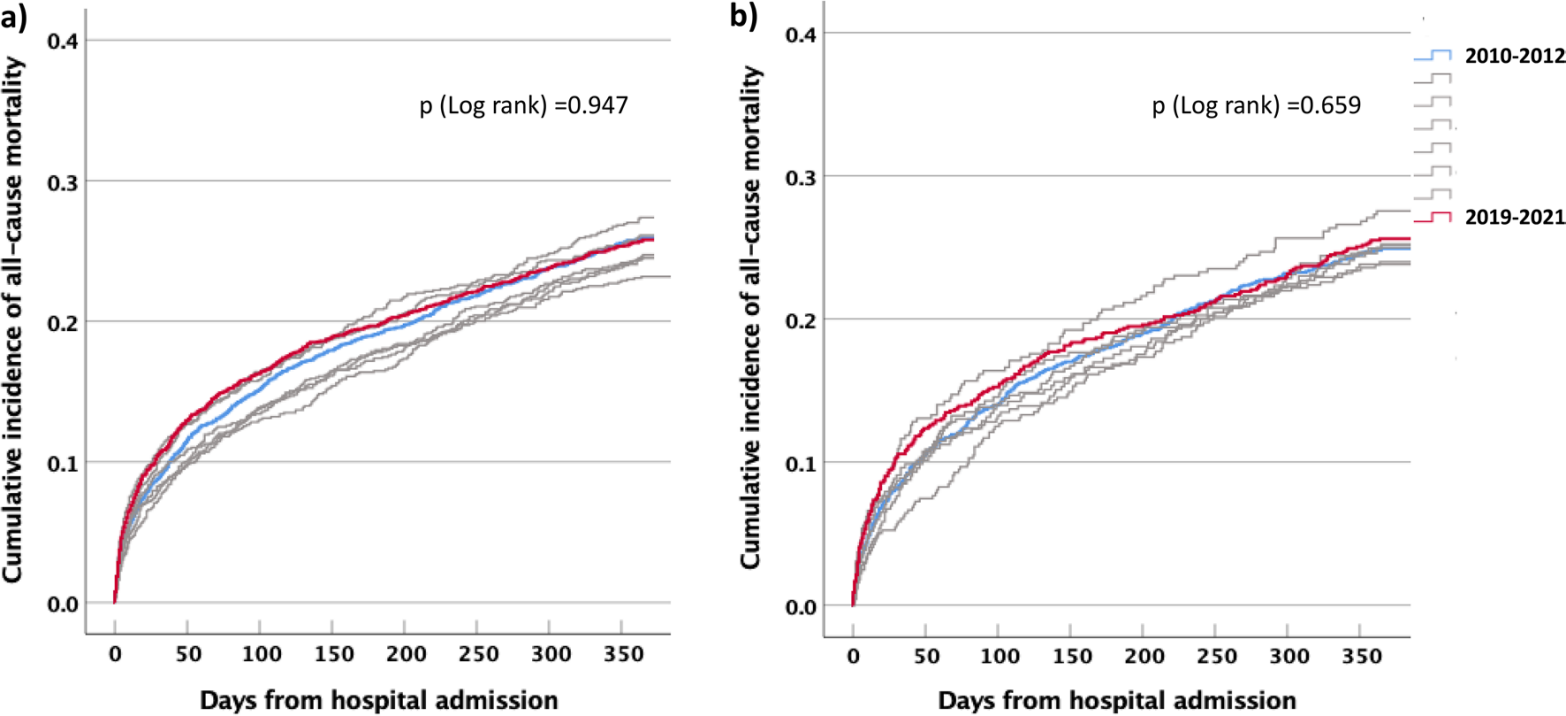
Cumulative incidence of all-cause mortality in patients with type 2 MI.(**a**) Total population; (**b**) patients with coronary artery disease. The shaded lines represent Kaplan–Meier curves for the years 2013–2019. P (Log rank) values refer to comparisons between patients admitted 2010–2012 vs. 2019–2021. *MI* myocardial infarction.

Information on all-cause mortality was available in 11,608 unique type 2 MI patients. A total of 2954 (25.4%) patients died within one year from admission. Mortality rates for patients admitted 2010–2012 were 26.0% (n = 748/2882), compared to 25.8% (n = 597/2313) for those admitted 2019–2021. The Kaplan–Meier curves yielded no clear pattern indicating an unidirectional mortality change in outcome over time (Fig. 2A and B). Upon multivariable adjustment, the admission years (2010–2012 vs 2019–2021) were neither associated with all-cause mortality in type 2 MI patients overall (odds ratio 1.00 [95% confidence interval 0.97–1.03]; *p* = 0.992) nor in those with CAD (odds ratio 1.03 [95% confidence interval 0.98–1.07]; *p* = 0.257). Similar findings were noted in men (odds ratio 0.99 [0.95–1.03]; *p* = 0.660) and women (odds ratio 1.01 [0.97–1.05]; *p* = 0.618) and in the sensitivity analysis restricted to patients aged < 80 years (odds ratio 1.01 [0.97–1.05]; *p* = 0.633).

## Discussion

The concept of type 2 MI was introduced in 2007 in the context of the publication of the Universal Definition^1^. Since then, type 2 MI has been recognized as a condition associated with a considerable burden of comorbidities and poor prognosis^2–7^. Paradoxically however, our data from Swedish type 2 MI patients admitted over a 11-year period (over 14 years from the publication of the Universal Definition) demonstrate lower use of diagnostic tests and lower medication provision without improvement over time. Moreover, we observed no major change in 1-year all-cause mortality. Our findings are intriguing, in particular since patients were managed by cardiology staff with a presumably high awareness on the clinical implications of type 2 MI.

Overall, we noted minor increases in the rates of echocardiography and coronary assessment. These changes however, were smaller compared to type 1 MI. Accordingly, they appear to represent rather a management change in the general MI population than improved routines in type 2 MI. The provision of RAAS-inhibitors and statins did not increase in the overall type 2 MI population including those with CAD, known to have the poorest prognosis^2,4^. This was in contrast to type 1 MI patients for whom interaction analyses indicated more favorable prescription trends. Prescriptions of betablockers decreased significantly in type 2 MI patients but remained at overall high levels, e.g. 82.5% in 2020–2022 in patients with CAD.

The lack of increases in prescription rates of cardioprotective medications in type 2 MI is intriguing. We can only speculate on possible reasons why physicians were reluctant to initiate such treatments. For some patients, the management of the triggering condition may have overshadowed the need of cardioprotective medications. Polypharmacy may have been an issue in other patients. Limited perceived life expectancy could have mattered as well since type 2 MI patients in general represent an aged population. However, the results from the sensitivity analyses restricted to patients < 80 years yielded at least similar decreases in prescription rates as noted for the entire type 2 MI cohort.

Sex-disparities in the management of type 2 MI may exist but evidence is still limited^14,15^. In a previous investigation from the SWEDEHEART registry, no indication of sex bias in the selection of type 2 MI patients for cardiac investigations was found^16^. We here extend these findings by demonstrating that temporal trends in medical interventions did not differ between women and men. However, we acknowledge that selection bias may be present since type 2 MI patients warded outside cardiology departments more often tend to be female^7^.

Our data emphasize the need of evidence-based care pathways in type 2 MI. Notably, these patients share many risk factors with those having type 1 MI^17^. It is thus, conceivable that aggressive risk factor management may achieve similar prognostic benefits in both MI types. Supporting evidence however, is scarce. Medication with statins has been shown to improve outcomes in MI patients without obstructive coronary arteries (MINOCA)^18^ among whom many may have had type 2 MI. Lipidlowering and antidiabetic treatments moreover, reduce the risk of future type 2 MI^19,20^. On a similar note, medications with betablockers or RAAS-inhibitors exhibit prognostic benefit in patients with type 2 MI^3^ and MINOCA^18^.

While there still are knowledge gaps on how to manage type 2 MI optimally, we believe that more intense diagnostic assessment and medication might improve outcomes in this vulnerable population. Recently published data suggest that the T2-risk score might facilitate the identification of high-risk type 2 MI patients with particular need for further investigation^21^. The Targeting Investigation and Treatment in Patients With Type 2 Myocardial Infarction (TARGET-Type 2) pilot study (NCT05419583, clinicaltrials.org) and the Appropriateness of Coronary Investigation in Myocardial Injury and Type 2 Myocardial Infarction (ACT-2) study^22^ will provide clarifying evidence in this regard. However, an agreement on optimal timing and the setting of cardiac assessment and follow-up in type 2 MI may be difficult since these patients represent a diverse cohort, often with complex health issues. We suggest that patients aged < 80 years should undergo cardiac assessment, either during the initial hospitalization or in the outpatient setting. Identified risk factors and cardiac conditions should be treated according to current guidelines. The underlying triggering condition should be managed by the respective specialty. We suggest individualized management approaches in patients aged ≥ 80 years depending on biological age and patient preference.

We acknowledge some study limitations that need to be considered. Our investigation is limited to a single healthcare system. Although all hospitals participating in SWEDEHEART are annually monitored, the data cannot be of the same quality as in a prospective study. However, the accuracy of the data and the registry has been found to be high^23^. The diagnosis of MI was set locally by the treating physicians. While the SWEDEHEART framework recommends the use of criteria outlined in the Universal Definition^9^, there was no independent adjudication. We can thus, not exclude erroneous diagnosis or subtyping of MI in some cases^24^. There may have been unmeasured confounders not documented in SWEDEHEART that could have influenced management decisions, e.g. patient refusal, comorbidities, frailty or short life expectancy. We lack information on the dosage of medications, examination results or treatment modifications performed after hospital discharge. Finally, our analysis is restricted to patients admitted to CCUs why selection bias may be present. Patients with type 2 MI are often given ward in other facilities with even lesser use of diagnostic procedures and medications according to recent Swedish data^7^. This implies the possibility of even greater management disparities compared to type 1 MI. Extrapolation of our findings to these patients should thus, be done with caution.

In conclusion, our data demonstrate no favorable trend in the use of diagnostic tests and provision of cardioprotective medications in patients with type 2 MI. This represents a missed opportunity to improve outcome. Accordingly, all-cause mortality in our study cohort remained largely unchanged during the observation period. Our findings thus, emphasize the need of defining optimal care pathways for type 2 MI patients.

## Supplementary Information

Supplementary Information.

## Data Availability

The data used in this study originates from the SWEDEHEART registry and contains sensitive patient information. The dataset analyzed in this study is not publicly available due to Swedish patient privacy, secrecy laws regulating access to SWEDEHEART, and due to ethical restrictions regarding the current analysis from the TOTAL-AMI project (Regional Ethical Review Board in Stockholm; reference number 2012/60-31/2). Data access can be made available at Uppsala Clinical Research Center upon reasonable request and under the provision that the data is accessed onsite and does not leave Uppsala University. This request can be sent to info@ucr.uu.se.
