# Supplementary material for: Management and outcome trends in type 2 myocardial infarction: an investigation from the SWEDEHEART registry
Source: Sci Rep. 2023 May 3;13:7194. doi: 10.1038/s41598-023-34312-7 (PMC10156703; doi:10.1038/s41598-023-34312-7)
Supplement: Supplementary file 1 — Supplementary Information. [file 41598_2023_34312_MOESM1_ESM.docx]

**Supplementary Data.**

**Supplementary Table S1. Temporal management changes in men and women with type 2 MI.**

**Supplementary Table S2. Temporal management changes in patients with type 2 MI – Sensitivity analyses.**

**Supplementary Table S3. Temporal changes in the provisions of cardioprotective medications in patients with type 2 MI without coronary artery disease.**

**Supplementary Figure S1. Annual proportions of MI patients with available information on MI type.**

**Supplementary Figure S2. Annual rates of A) echocardiography; B) coronary assessment.**

**Supplementary Figure S3. Annual rates in the provision of medications with**

**A) betablockers; B) RAAS-inhibitors; C) statins.**

**Supplementary Table S1. Temporal management changes in men and women with type 2 MI.**

|  | **Men** | | | **Women** | | |  |
| --- | --- | --- | --- | --- | --- | --- | --- |
|  |  |  |  |  |  |  |  |
|  |  |  |  |  |  |  |  |
|  | **n** | **OR (95% CI)** | **p-value** | **n** | **OR (95% CI)** | **p-value** | **p _interaction_** |
|  |  |  |  |  |  |  |  |
|  |  |  |  |  |  |  |  |
| **Examinations** |  |  |  |  |  |  |  |
| Echocardiography | 2614 | 1.09 (1.06-1.11) | <0.001 | 2628 | 1.06 (1.04-1.09) | <0.001 | 0.183 |
| Coronary assessment | 2327 | 1.07 (1.04-1.10) | <0.001 | 2351 | 1.06 (1.03-1.08) | <0.001 | 0.163 |
|  |  |  |  |  |  |  |  |
| **Discharge medications** | | |  |  |  |  |  |
| Betablockers | 2458 | 0.94 (0.91-0.96) | <0.001 | 2408 | 0.94 (0.92-0.97) | <0.001 | 0.779 |
| RAAS-inhibitors | 1419 | 1.00 (0.96-1.03) | 0.772 | 1468 | 0.95 (0.92-0.98) | 0.003 | 0.093 |
| Statins | 2495 | 1.00 (0.97-1.02) | 0.834 | 2463 | 0.99 (0.96-1.01) | 0.274 | 0.514 |
|  |  |  |  |  |  |  |  |

Multivariable logistic regressions were adjusted for hospital, age, current smoking, diabetes, hypertension, hyperlipidemia, congestive heart failure, previous myocardial infarction, previous percutaneous coronary intervention or coronary artery bypass grafting, previous stroke, atrial fibrillation upon admission, chronic obstructive pulmonary disease, previous or present cancer, peripheral vascular disease, estimated glomerular filtration rate and admission years (2010-2012 vs. 2020-2022).

P _interaction_ refers to the interaction of sex on the associations of admission years with medical interventions.

OR: odds ratio; CI: confidence interval; RAAS: renin-angiotensin-aldosterone-system.

**Supplementary Table S2. Temporal management changes in patients with type 2 MI – Sensitivity analyses.**

|  | **Admission years**  **2010-2012 vs 2020-2022**  **(age <80 years)** | | | **Admission years**  **2010-2012 vs 2020-2022**  **(first-time admission)** | | |
| --- | --- | --- | --- | --- | --- | --- |
|  |  |  |  |  |  |  |
|  |  |  |  |  |  |  |
|  | **n** | **OR (95% CI)** | **p-value** | **n** | **OR (95% CI)** | **p-value** |
|  |  |  |  |  |  |  |
|  |  |  |  |  |  |  |
| **Examinations** |  |  |  |  |  |  |
| Echocardiography | 3090 | 1.06 (1.04-1.08) | <0.001 | 4452 | 1.08 (1.06-1.09) | <0.001 |
| Coronary assessment | 2740 | 1.03 (1.01-1.06) | 0.003 | 3937 | 1.06 (1.04-1.08) | <0.001 |
|  |  |  |  |  |  |  |
| **Discharge medications** | | |  |  |  |  |
| Betablockers | 2940 | 0.92 (0.90-0.95) | <0.001 | 4117 | 0.94 (0.92-0.96) | <0.001 |
| RAAS-inhibitors | 1800 | 0.94 (0.91-0.97) | <0.001 | 2425 | 0.98 (0.95-1.00) | 0.061 |
| Statins | 2990 | 0.96 (0.94-0.99) | 0.002 | 4198 | 0.99 (0.97-1.01) | 0.410 |
|  |  |  |  |  |  |  |
| **Discharge medications in patients with CAD** | | |  |  |  |  |
| Betablockers | 1542 | 0.94 (0.90-0.98) | 0.002 | 1840 | 0.97 (0.93-1.00) | 0.086 |
| RAAS-inhibitors | 1028 | 0.92 (0.88-0.96) | <0.001 | 1209 | 0.97 (0.93-1.01) | 0.125 |
| Statins | 1571 | 0.94 (0.90-0.98) | 0.002 | 1880 | 1.00 (0.97-1.04) | 0.943 |
|  |  |  |  |  |  |  |

Multivariable logistic regressions were adjusted for hospital, sex, age, current smoking, diabetes, hypertension, hyperlipidemia, congestive heart failure, previous myocardial infarction, previous percutaneous coronary intervention or coronary artery bypass grafting, previous stroke, atrial fibrillation upon admission, chronic obstructive pulmonary disease, previous or present cancer, peripheral vascular disease, estimated glomerular filtration rate and admission year.

OR: odds ratio; CI: confidence interval; RAAS: renin-angiotensin-aldosterone-system; CAD: coronary artery disease.

**Supplementary Table S3. Temporal changes in the provision of cardioprotective medications in patients with type 2 MI without coronary artery disease.**

|  | **Crude data** | | | **Multivariable logistic regression results** | | | |
| --- | --- | --- | --- | --- | --- | --- | --- |
|  |  |  |  |  |  |  |  |
|  |  |  |  |  |  |  |  |
|  | **2010-2012** | **2020-2022** | **p-value** | **n** | **OR (95% CI)** | **p-value** | **p _interaction_** |
|  |  |  |  |  |  |  |  |
|  |  |  |  |  |  |  |  |
| Betablockers | 1010 (76.3%) | 731 (64.6%) | <0.001 | 2303 | 0.93 (0.90-0.95) | <0.001 | 0.093 |
| RAAS-inhibitors | 453 (74.4%) | 479 (73.6%) | 0.394 | 1231 | 0.99 (0.95-1.02) | 0.394 | 0.377 |
| Statins | 739 (54.5%) | 672 (58.4%) | 0.406 | 2344 | 0.99 (0.97-1.01) | 0.406 | 0.606 |
|  |  |  |  |  |  |  |  |

Multivariable logistic regressions were adjusted for hospital, sex, age, current smoking, diabetes, hypertension, hyperlipidemia, congestive heart failure, previous myocardial infarction, previous percutaneous coronary intervention or coronary artery bypass grafting, previous stroke, atrial fibrillation upon admission, chronic obstructive pulmonary disease, previous or present cancer, peripheral vascular disease, estimated glomerular filtration rate and admission years (2010-2012 vs 2020-2022).

P _interaction_ refers to the interaction of the presence of coronary artery disease on the associations of admission years with the provision of cardioprotective medications.

OR: odds ratio; CI: confidence interval; RAAS: renin-angiotensin-aldosterone-system.

**Supplementary Figure S1. Annual proportions of MI patients with available information on MI type.**

The annual prevalences of type 2 MI are presented within the respective bars. The respective total numbers of patients with available information on MI type are presented on top of each bar.

**Supplementary Figure S2. Annual rates of A) echocardiography; B) coronary assessment.**

| **A)** | **** |
| --- | --- |
| **B)** | **** |

Percentages refer to crude changes in the rates of diagnostic examinations (2010-2012 vs. 2020-2022).

MI: myocardial infarction.

**Supplementary Figure S3. Annual rates in the provision of medications with**

**A) betablockers; B) RAAS-inhibitors; C) statins.**

| **A)** | **** |
| --- | --- |
| **B)** | **** |
| **C)** | **** |

Percentages refer to crude changes in the rates of discharge medications (2010-2012 vs. 2020-2022).

RAAS: renin-angiotensin-aldosterone system; MI: myocardial infarction; CAD: coronary artery disease.
